# Method and software for using m-sequences to characterize parallel components of higher-order visual tracking behavior in *Drosophila*

**DOI:** 10.3389/fncir.2014.00130

**Published:** 2014-10-31

**Authors:** Jacob W. Aptekar, Mehmet F. Keles, Jean-Michel Mongeau, Patrick M. Lu, Mark A. Frye, Patrick A. Shoemaker

**Affiliations:** ^1^Department of Integrative Biology and Physiology, Howard Hughes Medical Institute, University of California, Los AngelesLos Angeles, CA, USA; ^2^Tanner Research Inc.Monrovia, CA, USA

**Keywords:** vision, optomotor, psychophysics, fixation, attention, *Drosophila*, system identification, dynamics

## Abstract

A moving visual figure may contain first-order signals defined by variation in mean luminance, as well as second-order signals defined by constant mean luminance and variation in luminance envelope, or higher-order signals that cannot be estimated by taking higher moments of the luminance distribution. Separating these properties of a moving figure to experimentally probe the visual subsystems that encode them is technically challenging and has resulted in debated mechanisms of visual object detection by flies. Our prior work took a white noise systems identification approach using a commercially available electronic display system to characterize the spatial variation in the temporal dynamics of two distinct subsystems for first- and higher-order components of visual figure tracking. The method relied on the use of single pixel displacements of two visual stimuli according to two binary maximum length shift register sequences (m-sequences) and cross-correlation of each m-sequence with time-varying flight steering measurements. The resultant spatio-temporal action fields represent temporal impulse responses parameterized by the azimuthal location of the visual figure, one STAF for first-order and another for higher-order components of compound stimuli. Here we review m-sequence and reverse correlation procedures, then describe our application in detail, provide Matlab code, validate the STAFs, and demonstrate the utility and robustness of STAFs by predicting the results of other published experimental procedures. This method has demonstrated how two relatively modest innovations on classical white noise analysis—the inclusion of space as a way to organize response kernels and the use of linear decoupling to measure the response to two channels of visual information simultaneously—could substantially improve our basic understanding of visual processing in the fly.

## Introduction

Visual figure detection is a central capability demonstrated by sophisticated visual systems, including those of flies (Reichardt and Wenking, 1969; Reichardt and Poggio, 1976). In some species, this capability extends even to tracking targets that subtend less than one ommatidial facet, and thus fall below classical detection limits (O'Carroll and Wiederman, 2014). Such sensitivity implies that figure tracking capitalizes on highly specialized neural mechanisms. On the basis of physiological studies in flies (Dipterans), cells housed by third and fourth-order visual neuropils in these animals—i.e., the lobula plate and lobula—are strongly implicated in such functions. Neural elements have been identified that have distinct responses to discrete visual objects, including “figure detecting” (FD) cells (Egelhaaf, 1985a,b), “small target motion detector” (STMD) cells (O'Carroll, 1993; Nordström et al., 2006; Nordström and O'Carroll, 2006), and even some lobula plate tangential cells (LPTCs) (Lee and Nordström, 2012) that for years have been supposed to serve primarily wide-field optic flow analysis. However, although progress has been made in understanding phenomenological aspects of figure detection in flies, its computational basis is still largely unexplained—as are the ways in which it relates to the various other perceptual modes of vision, and how they all are transformed and recombined or selected to produce calibrated motor commands for control of visual orientation.

**Figure 1:**
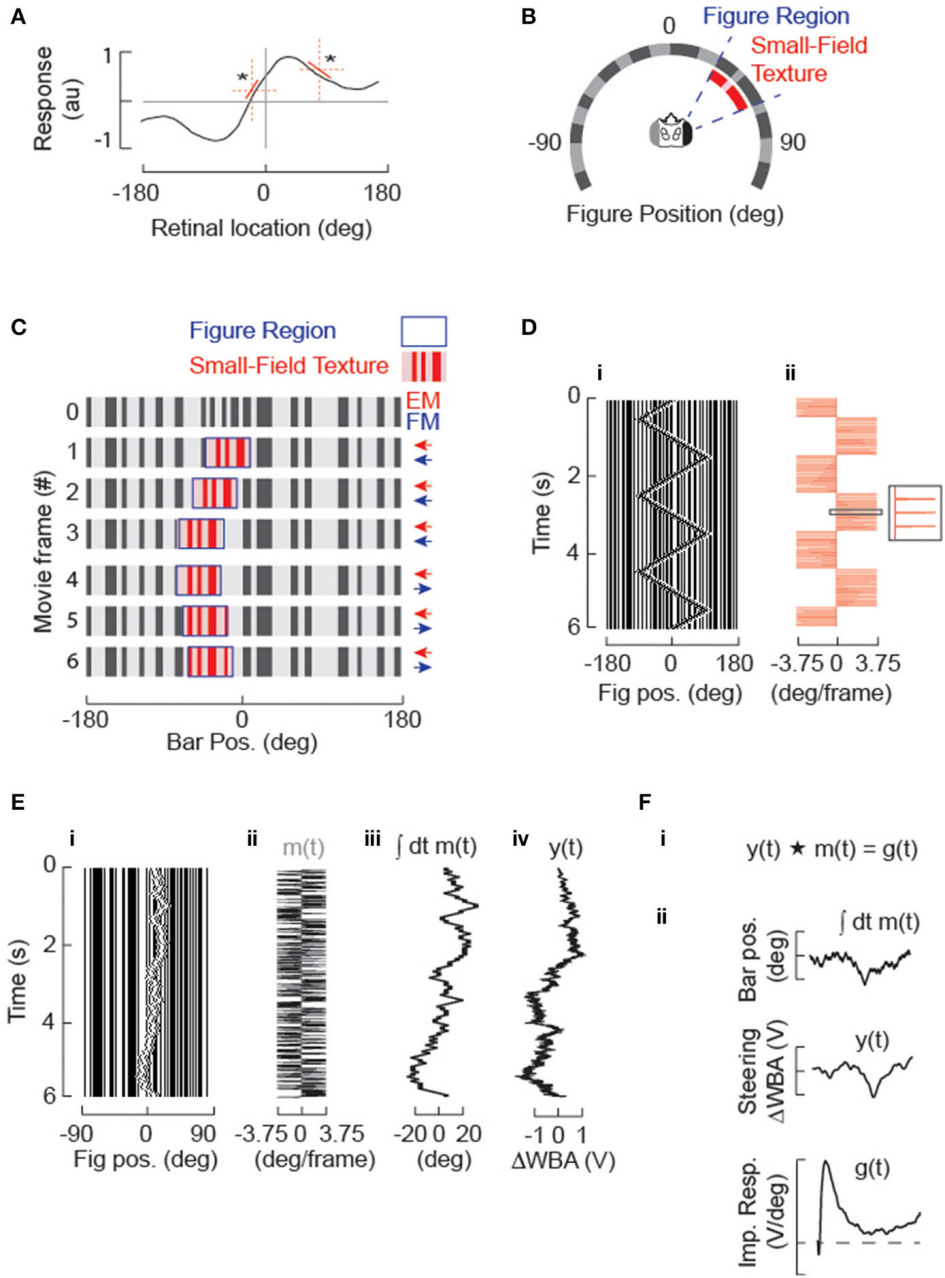
**Systems identification approach for studying figure tracking behavior. (A)** The amplitude of steering responses to arbitrary figure motion (or stationary flicker) may be non-linear over the visual field, (^*^ highlight two regions with different local rate of change in the dynamics of the steering response) but can be approximated over small spatial domains by a linear function (red). The STAF methodology approximates this steering response by estimating linear filters from m-sequences that are localized in space. **(B)** A circular display subtends 330° of the fly's visual field. The stimulus sequences are panoramic and 96 pixels in extent, but 8 physical pixels subtending 30° are omitted from the back of the display for access. A vertical grating of randomly segregated ON and OFF elements makes a stationary background containing broad band spatial wavelengths. A figure is defined by a 30° window (delineated in blue), within which the surface texture (denoted in red) varies from and replaces the background. The spatial statistics of the internal texture matches those of the background. The figure window itself can be displaced independently from the texture within it. **(C)** Example of figure motion. The figure is composed of the same pseudo-random pattern as the ground, therefore the figure is defined only by its relative movement. Displacement of the window provides figure motion (FM, highlighted in blue) that is undetectable by a standard motion detection model, which can be modulated independently from the displacement of the surface texture that generates small-field elementary motion that would be readily detected by an EMD-based system (small-field elementary motion [sf-EM] highlighted in red). In this simple case, a first-order “Fourier bar,” FM and sf-EM move coherently in the same direction for frames 1–3. In frames 4–6, FM is toward the right and there is no sf-EM within the figure window (i.e., the pattern within this window remains stationary). **(D)** A Fourier bar is displaced in one pixel steps 90° back-and-forth across the visual azimuth. (i) is a space-time plot of the stimulus (in which azimuth constitutes the only spatial dimension), and (ii) illustrates how that each 3.75° step (minimum pixel-spacing in LED arena) in the position of the figure corresponds to an impulse in velocity. **(E)** Motion of the solid Fourier bar (i.e., FM = sf-EM) is modulated by velocity impulses controlled by a m-sequence (see Methods) producing a pseudo-random motion trajectory centered in this case near visual midline. (i) Space-time plot of movie; (ii) m(t), pseudorandom sequence of impulse responses in velocity; (iii) position [time-integral of m(t)] of the figure; (iv) y(t), animal steering response to stimulus in (i). **(F)** Cross-correlation of the m-sequence (*m*) in degrees with the animal's steering response (*y*) proportional to the difference in amplitude across the two wings (Δ*WBA*) provides an estimate of the velocity impulse response (*g*).

In earlier work, a white-noise-based systems identification technique that is conventionally used with linear systems was applied to characterize the optomotor reactions of flies to various modes of wide-field motion (Theobald et al., 2010a). More recently, we have reported several studies of *visual figure tracking* in fruit flies (Aptekar et al., 2012; Fox and Frye, 2014; Fox et al., 2014) in which we elaborated on this basic technique to develop a representation known as the *spatiotemporal action field* (STAF). A *STAF* is defined as a *function of time and space* that represents a *temporal impulse response* for some behavioral reaction, evaluated as a function of the *position* of a feature in the visual field. It provides a dynamical model of optomotor behavior over some limited range of operating conditions. Its application to a (usually highly non-linear) biological system, like that supporting figure detection, requires an assumption of *local* or *quasi*-linearity (specified for those operating conditions), approximate time invariance (i.e., behavioral consistency), and *temporal superposition* of responses evoked at different spatial locations (Figure 1A). In order to be accepted as a dynamical model, it must be validated for the range of conditions over which it is supposed to be applicable. The aim of this paper is to promote understanding of the STAF methodology by describing the theory, the experimental context, and the analysis techniques surrounding the formalism in detail. In addition, we describe instances of its application to visual figure detection, including special measures taken to ensure its validity, the results so obtained, and finally provide relevant software and documentation to facilitate the use of the technique.

## Methods

### Application of the M-sequence technique to figure tracking in flies: dependence on figure and elementary motion

It has long been established that fruit flies will attempt to track—i.e., exert yaw torque to turn toward—to fixate—vertically elongated objects in their visual fields (Reichardt and Wenking, 1969; Maimon et al., 2008). The figure-centering fixation response in *Drosophila* is clearly seen for figures corresponding to actual physical objects– i.e., those in which the motion of any internal luminance patterns corresponds to the motion of the mean luminance distribution defining the object itself—but in addition to such first-order or *Fourier motion*, flies also track figures defined by the envelope of mean luminance (second-order) and also figures that are defined by higher-order properties that do not correspond to or do not contain first-order signals (Theobald et al., 2008; Aptekar et al., 2012; Zhang et al., 2013). For example, figures that comprise moving windows in which flickering patterns are displayed, or even figures in which the elementary or first-order motion of the internal texture is opposed to the direction of motion of the window itself [the so-called “theta” stimulus (Zanker, 1993)], all elicit a fixation response. The characteristics of the responses to these various types of figures do, however, differ measurably.

Based on prior experimental and theoretical work, it has been posited in the past that there are two components to figure tracking efforts: an optomotor response aligned with the velocity of motion, and an orientation response toward the position of flicker generated by motion (Reichardt and Wenking, 1969; Pick, 1976; Reichardt and Poggio, 1976; Wehrhahn and Hausen, 1980; Wehrhahn, 1981; Kimmerle et al., 2000). Recent evidence suggests that flies can in fact distinguish figures based on a broad range of spatiotemporal disparities, including cases in which flicker is uniform throughout the visual field (Theobald et al., 2010b), and our hypothesis with respect to figure tracking behavior is that the visual system of the fly extracts two streams of information in response to general figure motion, one related to the elementary motion of luminance edges of internal texture (small-field Elementary Motion, sf-EM), if present, and the other to the overall motion of the figure itself (Figure Motion, FM) under the assumption that the FM system encapsulates not only the position of local flicker (i.e., classical “position” system input), but also any other higher-order spatio-temporal statistical disparities generated by a *moving* figure., and that the total behavioral response approximates a superposition of efforts commanded by the two streams (Aptekar et al., 2012). In order to design practical experiments to test this hypothesis, a time-efficient and reliable assay methodology is needed. For this we use a technique based on the *maximum length sequence*, or m-sequence, which has proved to be a useful tool for linear time-invariant system identification. (For reference, the m-sequence technique and its mathematical underpinnings are reviewed in the Supplementary Material, Section 7.) We used m-sequence techniques to extract two independent, additive components—represented in terms of two functions, termed the “EM STAF” and the “FM STAF”– that together characterize visual behaviors in response to vertically-oriented moving figures.

The experimental context in which these concepts were studied (Aptekar et al., 2012) is illustrated in Figure 1. Details of the wingbeat analyzer, LED flight arena, control software, and data acquisition have been published previously (Reiser and Dickinson, 2008; Fox et al., 2014). All experimental and analysis scripts are freely available as Matlab code (see Supplementary Material). The visual figures used in all experiments were vertical bars or windows (subtending 120° vertically and 30° azimuthally in a fly's field of view), displayed against a static background in a cylindrical arena with the fly tethered at center (Figure 1B). The interpixel separation was 3.75°. Within the figure window was displayed a spatial pattern with the same spatial statistics as background. Motion of a Fourier bar, in which the EM and FM are identical, is illustrated in the first three frames of Figure 1C, whereas a presentation of FM with no EM (a “drift-balanced” stimulus) is displayed in frames 4–6. On the digital display, a triangle sweep of a Fourier figure (EM = FM, Figure 1Di) is produced by discrete velocity impulses that periodically reverse direction (Figure 1Dii). Figures 1E,F illustrate the application of the m-sequence technique. The figure is stepped one pixel in one direction or the other according to a periodically-applied m-sequence (Figure 1E) and the steering effort produced by the fly, quantified as the difference Δ*WBA* between left and right wingbeat amplitudes (Tammero et al., 2004), is measured and regarded as the system output *y*. If it is assumed that the responses to individual steps die out within the period of the m-sequence (an assumption to be examined in further detail below), circular cross-correlation of the output with the m-sequence can be used to obtain an estimate of a velocity impulse response or kernel function *g* (Figure 1F). This procedure relies on the fact that the autocorrelation of an m-sequence approximates a delta function (this approximation is imperfect due to the presence of a small dc error, as discussed in the Supplementary Material).

**Figure 2:**
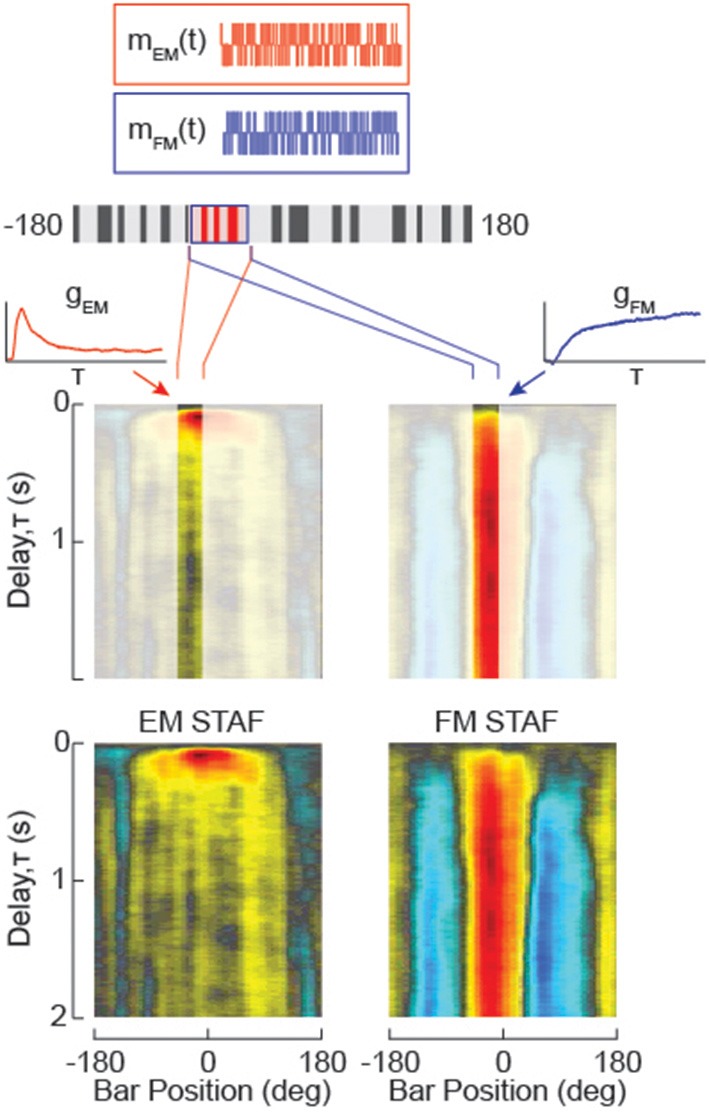
**Dissociating Figure Motion (FM) from small-field Elementary Motion (sf-EM) and measuring the non-linear variation in the impulse response to the motion of each over space**. Two m-sequences (*m*) are used to independently modulate the elementary motion of the small-field surface of the figure (sf-EM, red) and figure motion (FM, blue). FM in the absence of sf-EM would resemble a drift-balanced figure in which the figure “overwrites” the ground pattern with a new random texture, but generates no coherent motion signals. A property of the m-sequence is that the figure ends the trial displaced one pixel from its starting location, and the mean position is centered on the starting location. Cross-correlation of each of the two m-sequence signals with the difference of left and right wingbeat amplitude (ΔWBA) steering response data yields two impulse response estimates for the sf-EM stimulus (*g*_*EM*_(*t*)) and the FM stimulus (*g*_*FM*_(*t*)). By evenly sampling the visual azimuth of the LED display, the impulse response filters are concatenated into a function of space and time, a spatio-temporal action field (STAF) for the sf-EM and FM signals, respectively (at bottom). These functions are spatially smoothed with a four pixel boxcar.

There is ample evidence that magnitudes of reactions to first-order motion (Krapp et al., 1998) and to figures (Pick, 1976; Reichardt and Poggio, 1979) vary with stimulus location in the visual field. The STAF representation that characterizes such variation is therefore constructed by applying the stimuli around the entire visual field in the azimuthal direction. Because m-sequences are non-stationary and applied periodically, the required cross-correlations can be performed over sliding windows at various azimuths, each containing one full period of the m-sequence. The spatial dependence of the system is assumed to be approximately linear over the corresponding narrow range of figure positions, as illustrated in Figure 1A. A kernel function *g*(*t*) extracted from a single period is associated with the average position of the figure centroid during the period, and the set of kernel functions for all such positions are *concatenated* to obtain a STAF representation (Figure 2). The STAF is therefore defined at discrete times *t* (i.e., at multiples of the sampling interval) and discrete azimuth angles γ (the mean locations assumed by the figure centroids over the various individual m-sequences). With respect to the spatial resolution of this scheme, it can be shown that if the position dependence of a kernel function is approximately linear over the range of figure positions assumed during a single cycle of the m-sequence, then the estimate of the kernel computed over that cycle is very nearly equal to its value at the average position. In order to ensure that this was the case, we used relatively short m-sequences (of length *p* in the range 127–255) so that the total excursion of the figure was limited during any single period. For example, the standard deviation of the displacement from the mean position for a 7th order (127 element) m-sequence is between 3 and 4 pixels, or about 15°. The STAFs obtained for several sequence lengths were compared to verify that the spatial dependence was captured at these lengths.

In the primary set of experiments reported in Aptekar et al. (2012), we used a compound stimulus, in which the position of the figure window and the spatial texture internal to the figure were stepped *independently* at the same times—the figure according to one m-sequence *m*_*FM*_, and the internal pattern according to a second *distinct* m-sequence *m*_*EM*_ of the same order, as suggested in Figure 2. Under the hypothesis that EM- and FM-driven components of the response are quasilinear and they superpose, two independent kernel functions, *g*_*FM*_(*t*) for figure motion and *g*_*EM*_(*t*) for internal elementary motion, can be obtained by cross-correlation of the output with *m*_*FM*_ and *m*_*EM*_, respectively. The function *g*_*FM*_(*t*) represents the impulse response with respect to figure velocity, i.e., $\dot{\gamma }$, and *g*_*EM*_(*t*) the impulse response with respect to the velocity *v*_*EM*_ of the internal first-order motion. In addition to the autocorrelation property of m-sequences, this analysis relies on the fact that the cross-correlation of distinct m-sequences is nearly zero (see the Supplementary Material). The *g*_*FM*_ and *g*_*EM*_ obtained at different locations may each be concatenated around the azimuth to obtain respective STAF representations *G*_*FM*_(*t*, γ) and *G*_*EM*_(*t*, γ), as illustrated at bottom in Figure 2.

The Fourier transforms of these STAFs, according to the customary linear time-invariant systems interpretation, would give the frequency-domain representation of the system transfer functions parameterized by azimuth. These may be useful for qualitative characterization of the STAFs (e.g., how they may be interpreted as filters), but due to the restrictions discussed in below, they cannot be interpreted as general models of the optomotor figure response.

The most basic restriction on STAFs as models relates to the limits of quasilinear behavior of the system relative to the experimental protocols used to determine them. When stimuli conform to such limits, then under the assumption of temporal superposition, a STAF-based model for the optomotor figure response can be expressed in the time domain in terms of convolutions of the position-dependent kernels *G*_*FM*_(*t*, γ) and *G*_*EM*_(*t*, γ) with, respectively, azimuthal figure velocity $\dot{\gamma }$ and the velocity *v*_*EM*_ of elementary motion (if present). In these time-domain convolutions, figure positions must be parameterized according to the times at which they were assumed. If motion begins at time *t* = 0, then the complete expression for the steering response is:

(1) $$\begin{array}{l} y(t)=\int_{\tau \;=\;0}^{t}[G_{FM}\left(t-\tau ,\gamma (\tau )\right)\cdot \dot{\gamma }\left(\tau \right)\;\\ \;+G_{EM}\left(t-\tau ,\gamma \left(\tau \right)\right)\cdot v_{EM}(\tau )]d\tau \\ \;+\int_{\theta \;=\;0}^{\gamma (0)}G_{FM}\left(t,\theta \right)d\theta . \end{array}$$

The origin for the azimuth angle γ is identified with the figure location at which no steering effort is exerted by the FM system, that is, at front center of the animal. The second integral term in (1) represents the effect of the initial figure position as predicted by this model; it is zero if the figure starts at front center. The response of the FM system to a *stationary* figure at azimuth γ predicted by the model would be $\int_{\theta \;=\;0}^{\gamma }G_{FM}\left(\infty ,\theta \right)d\theta$.

In practice, the STAF estimates are computed (that is to say, *sampled*) only at discrete times and positions. The STAFs obtained in our study (Aptekar et al., 2012) vary smoothly and could be interpolated to obtain values off of this sampling grid when dealing with continuous motion, or with discrete time and position grids differing from the original. In point of fact, most laboratory display technologies produce sequences of discrete image frames and will thus impose position steps/velocity impulses at discrete times. In such case, the convolution in (1) becomes a sum:

(2) $$\begin{array}{l} y\left(t\right)=\sum_{\tau \;=\;0}^{t}G_{FM}\left(t-\tau ,\gamma (\tau )\right)\cdot \Delta_{FM}\left(\tau \right)\\ \;+G_{EM}\left(t-\tau ,\gamma \left(\tau \right)\right)\cdot \Delta_{EM}(\tau )\\ \;+\;\sum_{\theta \;=\;0}^{\gamma (0)}G_{FM}\left(t,\theta \right)\cdot \Delta_{F}\left(\theta \right) \end{array}$$

where Δ_*FM*_(τ) represents the step in figure position and Δ_*EM*_(τ) the step in internal pattern position at discrete time τ over the particular stimulus history, and Δ_*F*_ is the magnitude of the figure step at each discrete angle θ for which *G*_*FM*_(*t*, θ) is defined between θ = 0 and θ = γ(0).

### Experimental design: special measures for figure tracking

With this approach, care must be taken to consider likely deviations from linearity and other effects that influence the interpretation of the STAF as characterizing the optomotor system, and to ensure this, a number of special measures were taken in the design of experiments and analysis of the resulting data.

For instance, there is a great deal of evidence that elementary motion is processed in the visual system by local elementary motion detectors (EMDs) that compute spatiotemporal luminance correlations between neighboring or nearby visual sampling units (Buchner, 1976; Egelhaaf et al., 1989; Haag et al., 2004). Because the EM STAF depends on first-order motion, it is reasonable to assume that the neural machinery underlying it must involve EMDs. The operation of the EMD is inherently non-linear, and its output depends not just on velocity of motion but other characteristics of the visual scene as well. However, areas of visual texture in our experimental protocols conform to consistent spatial statistics, and when they move they are stepped at a regular rate by a single pixel, which is on the order of the inter-receptor angle—so under these conditions it may be justifiable to interpret the mean EMD response to an individual step as an impulse response function. We also expect that if a number of EMD outputs were summed over a region of retinotopic space, such as the area subtended by a finite-sized object, there would be a relative reduction in the standard deviation of the resulting signal. If the downstream processing that transforms the summed outputs into a motor command is approximately linear, then the interpretation of a *behavioral* step response may be justifiable. Prior results suggest that this is indeed the case for optomotor responses to wide-field motion (Theobald et al., 2010b).

However, from this qualitative discussion it is clear that constraints must be imposed on the design of experiments used to determine a STAF that depends on EMD processing—and similarly, that limits apply to interpretation of the results. For one, motion impulse responses ought not be estimated based on object steps much greater than the spatial basis of the EMD correlation; the variance of the output increases while its expected value approaches zero as the longest spatial wavelengths in the image are exceeded by the step. In addition, because the dependence of mean EMD output on image speed is non-linear (and in fact non-monotonic), the accuracy of an EM STAF as a representation of the optomotor control system is likely to degrade as object speeds vary significantly from the product of the step size and image update rate used in its experimental determination.

Currently, little is known about the processing that enables the fly visual system to distinguish a figure from background based on the variety of spatiotemporal differences that have been shown to support figure tracking in behavioral experiments. Thus, there is no guidance available from computational theory about the limits of an experimentally-determined FM STAF as a representation for optomotor behavior. However, one result of prior studies is especially significant with respect to its estimation: as mentioned in the prior section, there is a component of figure response that both theory and experiment suggest is fundamentally *position-dependent* (Pick, 1974; Buchner et al., 1984), in that steering efforts can persist for seconds when the position of a figure is *stationary* and it is located away from front center in the visual field (Pick, 1976). It is not known at present if this effect can be well-represented as the asymptotic behavior of an FM STAF obtained from experiments with *moving* figures. However, we should at least expect that reactions of the FM system to steps in figure position may be more akin to *step* than *impulse* responses, in that (unlike an EM-dependent STAF) they may assume non-zero values at long times. In order to extract the kernel associated with the figure response, we assumed that the *slope* of such position step responses does approach very small values over times corresponding to the duration of one cycle of an m-sequence, and made use of the fact that the time derivative of the output in response to figure motion can be written:

(3) $$\frac{dy}{dt}=\frac{d(m_{FM}*g_{FM})}{dt}=m_{FM}*\frac{dg_{FM}}{dt},$$

where ^*^ indicates temporal convolution. In this case, the cross-correlation *u*_*FM*_ of *dy*/*dt* with *m*_*FM*_ may be computed to provide an estimate of the derivative *dg*_*FM*_/*dt* of the desired kernel function, and this may (in principle) be integrated to obtain *g*_*FM*_. However, the *dc error term* also present in this cross-correlation, when integrated, would result in an accumulating error that would nearly cancel the desired result at times approaching the duration, *t* = *p* − 1 of the m-sequence. Thus, it is desirable to take measures to correct for this dc error. We note that this error, which takes the value $-\frac{1}{p}\sum_{j=0}^{p-1}\frac{dg_{FM}}{dt}$, is proportional to the asymptotic value of *g*_*FM*_ at long times, and thus may be eliminated if this asymptote can be estimated and added to *u*_*FM*_ prior to integration. For this purpose, we use the average of $\sum_{j\;=\;0}^{k}u_{FM}$ over times *k* corresponding to 2–5 s. During this interval the slope *dy*/*dt* typically assumes small values. Formally, this approximates the DC response term as having the same magnitude as terms for very low bar velocity, for which there is no measurable deviation of the steering effort from the static bar position, consistent with the fly tracking the absolute position, rather than the very low velocities of the bar.

It should be emphasized that the contribution to *G*_*FM*_(*t*, γ) obtained by integration of (3) at a particular γ represents the *change* in the FM-driven figure response induced by a step in figure position at that location—i.e., the FM STAF is an *incremental* representation.

When elementary motion is present within the figure, the analysis of *its* contribution is complicated by the figure position response: if *g*_*EM*_ is estimated by cross-correlation of *m*_*EM*_ with *y*, the estimate is contaminated by the dc component of the FM response whenever the figure is off of the midline. In addition, when relatively short m-sequences are used (as was the case in our experimental design), the cross-correlation between *m*_*FM*_ and *m*_*EM*_ may also be appreciably different from zero. This results in cross-contamination of the estimates for both *g*_*FM*_ and *g*_*EM*_; that is, each would be the sum of the desired kernel and a small proportion of the other when a simple cross-correlation is used. In order to reduce these sources of error, our full protocol comprised two sets of stimuli, interleaved randomly in time and each covering the entire visual field. In one, *m*_*FM*_ and *m*_*EM*_ respectively drove the figure and internal pattern steps, whereas in the second, *m*_*FM*_ and −*m*_*EM*_ were used. The outputs in these two cases are, respectively,

$$\begin{array}{l} y_{1}=m_{FM}*g_{FM}+m_{EM}*g_{EM},\\ y_{2}=m_{FM}*g_{FM}-m_{EM}*g_{EM}. \end{array}$$

During analysis, an estimate of *g*_*EM*_ can be formed by cross-correlating *m*_*EM*_ with the *difference* between these two output sequences (or equivalently, taking the difference between the cross-correlations with each):

(4) $$2u_{EM}=m_{EM}*y_{1}-m_{EM}*y_{2},$$

in theory eliminating the effect of the dc figure position response as well as any cross-contamination due to finite cross-correlation. Similarly, the *sum* of cross-correlations of the derivatives of the output sequences with *m*_*FM*_ yields a cross-contamination-free estimate of *d*(*g*_*FM*_)/*dt*:

(5) $$2u_{FM}=m_{FM}*dy_{1}/dt+m_{FM}*dy_{2}/dt,$$

where the use of the dc error correction methodology discussed above is implicitly assumed.

Due to the nature of the compound stimulus, one additional and subtle source of cross-contamination between the kernel estimates is present. When the figure and internal pattern are stepped syndirectionally in our protocol, the entire 8-pixel-wide pattern is shifted by one pixel in the common direction of motion, and there is the potential for spatiotemporally-correlated changes across 8 interpixel boundaries. However, when the two are stepped antidirectionally, only the center 6 pixels of the internal pattern are visible before and after the step, so that spatiotemporally-correlated changes can appear only across six boundaries. Thus, the effective extent of the coherently moving pattern is larger for syndirectional motion, and we would expect the response component driven by elementary motion to also be larger than for antidirectional steps. The stimulus used in practice was therefore modified to eliminate this source of cross-contamination by replacing the boundary pixels of the figure at random for each syndirectional step in the entire sequence.

Finally, a related issue with short sequences is the presence by sheer chance of more spatiotemporal correlations in one direction than the other during a cycle of the sequence, as a figure passes over and the fixed background becomes visible. In order to reduce this effect, we replaced the random background pattern every three periods of m-sequence excitation during the course of an entire experiment.

In our study, the magnitudes of the figure and elementary motion steps were 3.75° for all applied stimuli (although the signs of each of course varied with time in a manner unique to each stimulus). In any event, the validity of this representation should be expected to hold only for circumstances in which the mean velocity of motion approximates the product of the step size and image update rate used in the experimental determination of the STAFs.

## Results

### Application to tracking of general figures in drosophila

The results of our original figure tracking study using white noise techniques support the hypothesis that the total response to a figure against a static background approximates a superposition of efforts commanded by two processing streams, as characterized by the EM and FM STAFs (Aptekar et al., 2012). The spatial and temporal characteristics of the two STAFs differ significantly. The temporal dependence of the EM-STAF shows a clear “impulse-response” shape, with a short onset delay, rapid integration time, and near-zero asymptote, consistent with response to the velocity of the EM. In contrast, the FM-STAF displays a slow onset delay and persists for many seconds, consistent with a slower effort to track the retinotopic position of the figure. The EM response is strongest when the figure is present within the frontal field of view, diminishing gradually in amplitude with increasing displacement of the figure away from midline. In contrast, the spatial profile of the FM-STAF resembles a classic “center-surround” function in that the peripheral response is inverted relative to the response at the midline, and the spatial integral over the entire azimuth is near zero. This indicates that an incremental change in figure position within the frontal field of view results in an increment in the steering effort toward the figure (positive gain), but a position step within the periphery results in a decrement in the steering effort (negative gain, although not necessarily a reversal in the steering direction since the STAF is an incremental representation). Furthermore, our experiments confirmed that the FM system can operate in the absence of any coherent motion. We presented a moving figure that was dynamically updated with a new random internal pattern at each time step, such that no net coherent motion was present in the stimulus in any direction. When the motion of such a figure is driven by a single white noise sequence, the spatial and temporal characteristics of the turning reactions and the derived STAFs are nearly identical to those of the FM-STAFs obtained from the original figures containing uncorrelated EM. Furthermore, for a stimulus in which EM and FM of the figure covary (i.e., a Fourier bar), the resultant STAF is, to good approximation, simply the sum of the FM-STAF and EM-STAF obtained from the original experiment.

### Validation of assumptions and models

#### Response to standard figure stimuli

**Figure 3:**
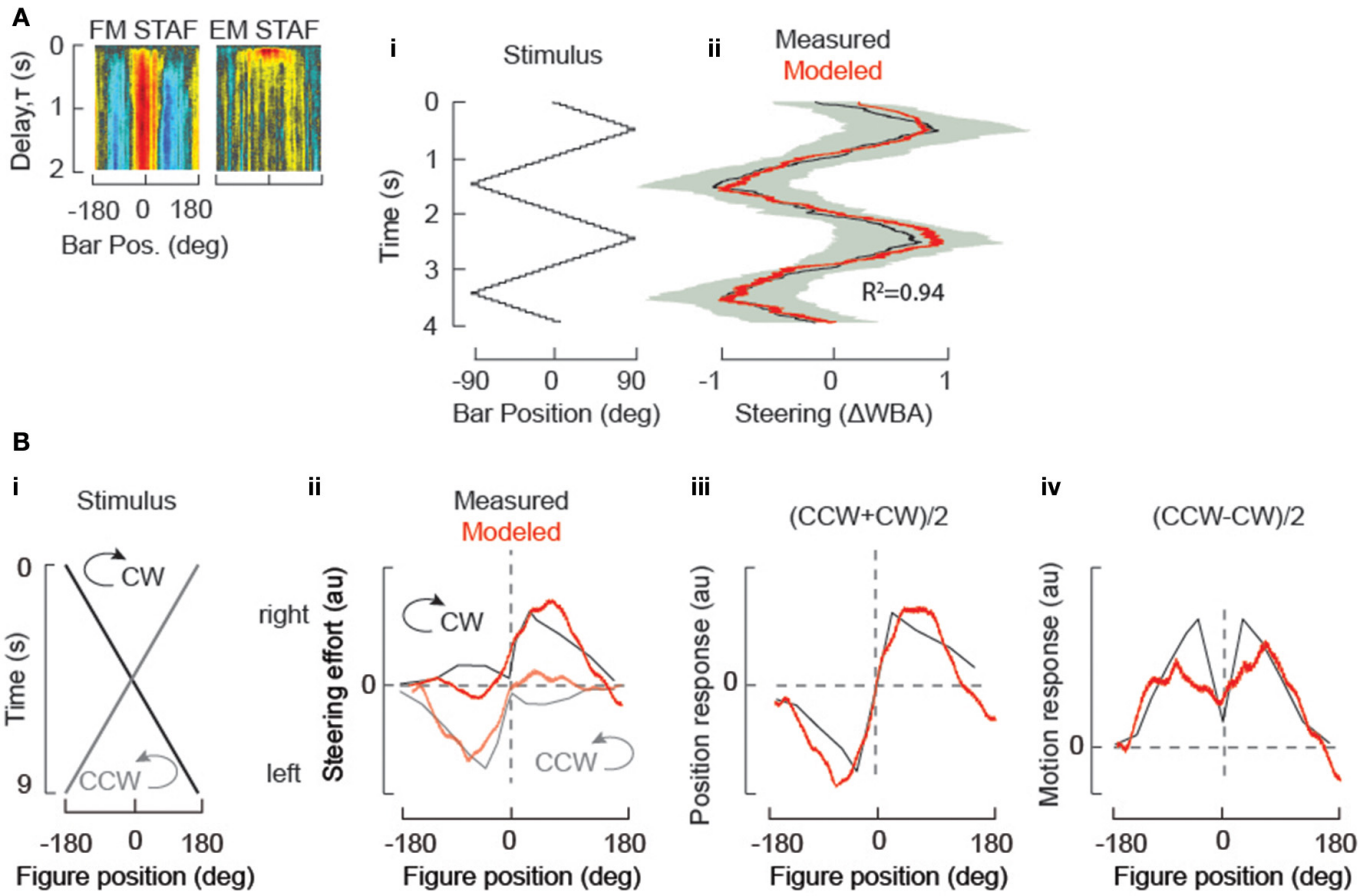
**STAF validation: STAFs predict steering responses to simple periodic stimuli. (A)** (i) “trisweep” trajectory of a solid Fourier bar. (ii) measured responses of wild-type flies (black indicating mean of *N* = 15 flies, and s.e.m. indicated by gray shaded envelope), and responses predicted by convolution of trisweep trajectory with both of the sf-EM and FM STAFs (indicated with insets at left) (red). R^2^ = coefficient of determination, indicating degree of correlation between STAF estimate and actual behavior. **(B)** STAFs predict responses measured under different experimental conditions. (i) the stimulus trajectory of a bar revolving around a circular arena at constant velocity in either the clockwise (CW) or counterclockwise (CCW) directions. (ii) convolution of the stimulus trajectory from (i) with both the sf-EM and FM STAFs models to predict turning responses (red). Overlaid (gray) are the mean steering responses to CW and CCW rotation of a dark bar presented to a fly tethered upon a floating ball (data reproduced from Bahl et al., 2013, Figure 2), and similar to results from Reichardt and Poggio (1976). Note that STAFs were measured in flight, and the data were measured from walking flies, so to facilitate comparison we normalized the steering responses and STAF predictions. (iii) for a sufficiently slow stimulus, addition of the bi-directional fly turning responses to the revolving bar produces an estimate of turning response to the bar's position (gray), which is well-approximated by the addition of the two STAF predictions from (ii) (red). (iv) subtraction of the bi-directional fly turning responses to the revolving bar produces an estimate of the turning response to the local motion of the bar (gray), which is well-approximated by the subtraction of the two STAF predictions from (ii) (red).

The most authoritative and general validation of the STAF-based model is its predictive power with respect to arbitrary stimulus scenarios. In Aptekar et al. (2012), we predicted responses to triangle sweeps of Fourier bars, of theta bars (in which the EM of texture within the bar is opposite in direction to the FM), and to trajectories in which EM and FM were driven by novel independent m-sequences (i.e., sequences different than those used to obtain the STAFs). During these simulations, the EM and FM step magnitudes and update rates were maintained at the same values as in the experiments used to determine the STAFs, and responses were predicted as the superposition of EM and FM responses as in (1). Predictive power was assessed by computing Pearson's R^2^-values for modeled vs. experimentally measured responses to these stimuli—and was found to be 0.9 or greater in all three cases. We have reproduced one such comparison for a Fourier figure sweeping at constant velocity across the frontal 180° of the visual field (Figure 3Ai). Measured and STAF-modeled results are in very close agreement (Figure 3Aii, with the STAFs used for the model indicated within the inset at left).

#### Response symmetry

A corollary of our assumption of quasilinearity is that the responses to progressive and regressive motion (either EM or FM) at a given velocity are roughly equal and opposite in sign at any location in the visual field. While the white noise technique captures the first-order component of behavior, i.e., the first-order Volterra kernel, even when non-linearity is present, the accuracy of the STAF as a dynamical model depends on how well linearity is approximated. However, results from other studies have been interpreted as suggesting that such asymmetry is in fact present. For example, Bahl et al. (2013) postulate that figure responses can be decomposed into “position” and “motion” components (roughly comparable to our FM and EM responses, respectively) and attempted to isolate these components in two distinct experiments. Discrepancies between the results of these experiments were taken as evidence for response asymmetry in that study.

To examine this issue, we considered the results of this prior study (Bahl et al., 2013), which addressed the cellular mechanism of EM detection for figure tracking by a tethered fly walking on an air-supported ball. In such an experiment, the fixed fly can “steer” the ball by walking in different directions. The apparatus is surrounded by several computer monitors that project perspective-corrected revolutions of a solid black vertical bar on a white background. The bar was rotated at constant velocity, and the fly's turning effort was measured by the displacement of the ball below the tethered fly. In response to constant velocity revolution of the bar in each of two directions (clockwise and counter clockwise), the animals tend to show smaller responses to the bar as it revolves from the rear toward the frontal field of view (back-to-front, BTF) by comparison to the steering response when the bar crosses midline and moves front-to-back, FTB). We used the STAFs collected from *flying* animals to predict the responses of the *walking* flies. Convolving the stimulus trajectory (Figure 3B) with the EM and FM STAFs (Figure 3A inset) produces modeled estimates that qualitatively match the behavioral responses of walking flies plotted in Bahl et al. (Figure 3Bii). To estimate the response component generated by the static position of the bar, Bahl et al. added the CCW and CW spatial trajectories, which are well approximated by our STAF predictions (Figure 3Biii). To estimate the response component generated by the motion of the bar, Bahl et al. subtracted the spatial trajectories. This predicts that the fly's response to elementary motion is at a minimum for an object in the frontal visual field, which directly opposes the prevailing evidence in the field. However, our STAF predictions show that this phenomenon is not a result of insensitivity to motion in the frontal visual field because we can recapitulate this apparent result using our STAFs which show maximal sensitivity to EM and FM in the frontal visual field (Figure 3Biv).

We conclude that the result observed by Bahl et al., was accentuated by a stimulus that moved at a rate that maximizes the apparent effect of hysteresis on the fly's steering behavior. We then show that the same effect is observed when the stimulus from Bahl et al., is convolved with our STAFs. We concede that it may be surprising that the results would be so similar for walking and flying animals, but argue that this explanation is more parsimonious than the unexpected alternative that walking flies are relatively insensitive to frontal motion (i.e., a prominent dip in the motion response function for a figure positioned near 0°, Figure 3Biv).

Hence, the STAF functions provide robust predictions of figure tracking responses to arbitrary visual stimuli presented in the same behavioral apparatus in which the STAFs were measured (Figure 3A), as well as qualitatively reasonable approximations to behavioral measures taken with walking flies in a completely different apparatus (Figure 3B).

**Figure 4:**
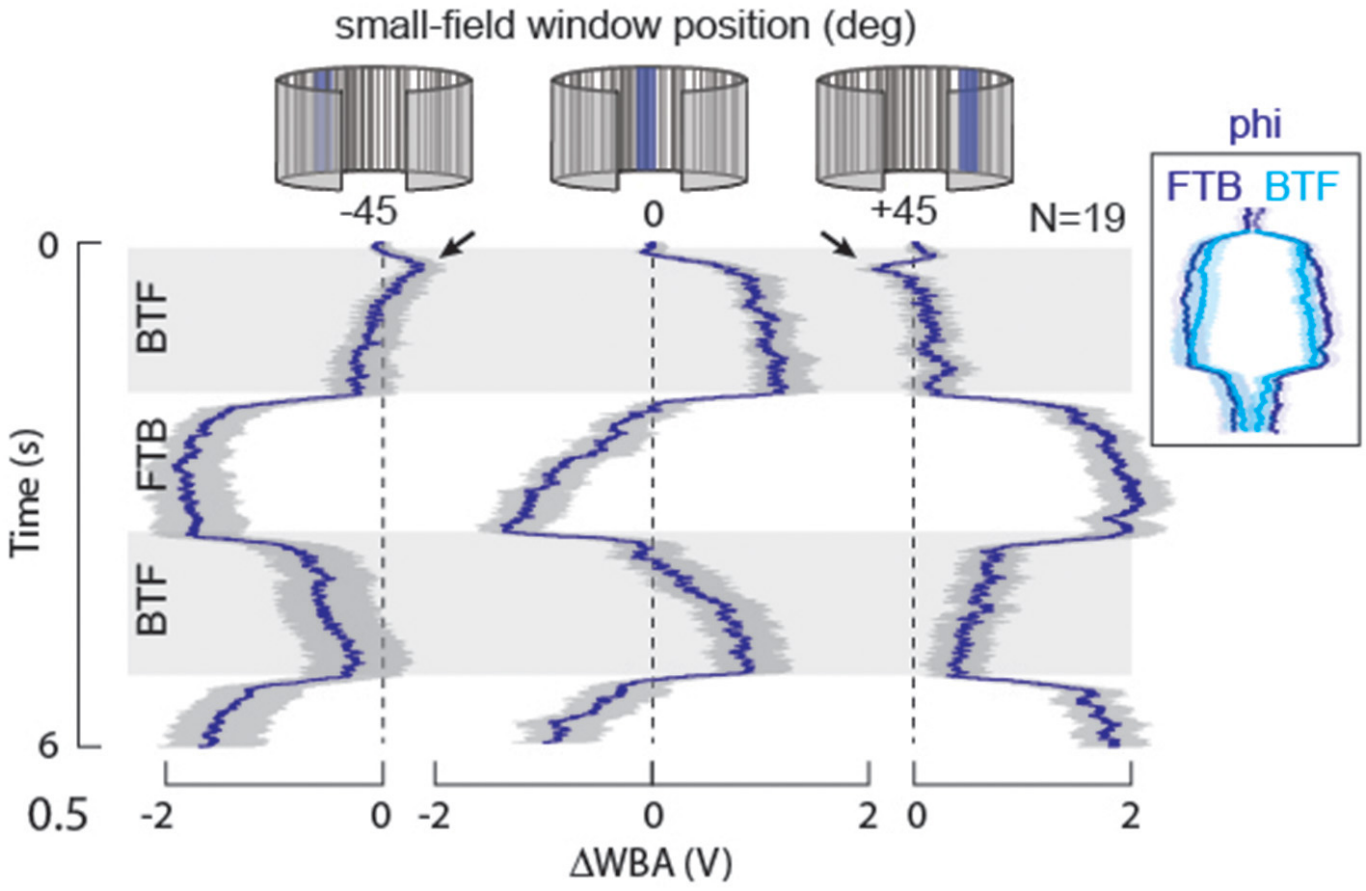
**STAF validation: motion response symmetry**. We presented small-field motion within a stationary 30° wide window (EM only, no FM) positioned either directly in front of the fly, or displaced 45° laterally on either side. Mean responses indicated in heavy lines, s.e.m. indicated with shading. Note that the motion-induced responses are nearly symmetric, and opposite for reverse-phi. BTF, indicates regressive, back-to-front motion on the eye; FTB, indicates progressive, front-to-back motion. For the stimulus at ±45°, the rapid motion-induced oscillations are superimposed upon a slow DC turn toward the window. Arrowheads indicate onset transients in which the fly briefly steers in the direction of the motion stimulus, and then slowly steers opposite, toward the position of the small-field window. Inset: the FTB response at −45° is superimposed upon the following BTF response, which has been reflected about the vertical axis, to demonstrate that the time course and steering trajectory of FTB responses in this case are nearly equal and opposite to the BTF responses.

Based on these results, we conclude that response asymmetry occurs for figure motion along extended continuous paths, and is a consequence of the spatial variations of the response characteristics in combination with their temporal dependence. Small displacements, conversely, do not produce the asymmetry. This view is supported by results of studies on these animals under stimulus conditions similar to ours (Buchner, 1976; Reichardt and Poggio, 1979; Kimmerle et al., 2000; Maimon et al., 2008; Theobald et al., 2010b). One example appears in Figure 4B of Maimon et al. (2008), in which a solid dark bar was oscillated about several mean positions relative to the visual midline. The fly's steering response has two components: a slow sustained turn toward the bar's position when it is off the midline, and a superimposed oscillatory steering response. At every mean azimuth for which the periodic response is significant, it is symmetric; there is no clear evidence of the pronounced harmonic distortion that would result from significant asymmetry between front-to-back and back-to-front responses. Similar results are obtained from experiments in our own lab (Figure 4). By way of comparison, asymmetry is apparent in experiments using longer trajectories (Götz, 1968; Reichardt and Poggio, 1976; Maimon et al., 2008; Bahl et al., 2013). Both sets of findings are valid, but the key finding with respect to our work is that the STAF model is capable of capturing extended-path results.

#### STAFs predict reverse-phi illusion for wide-field yaw, but not small-field EM

**Figure 5:**
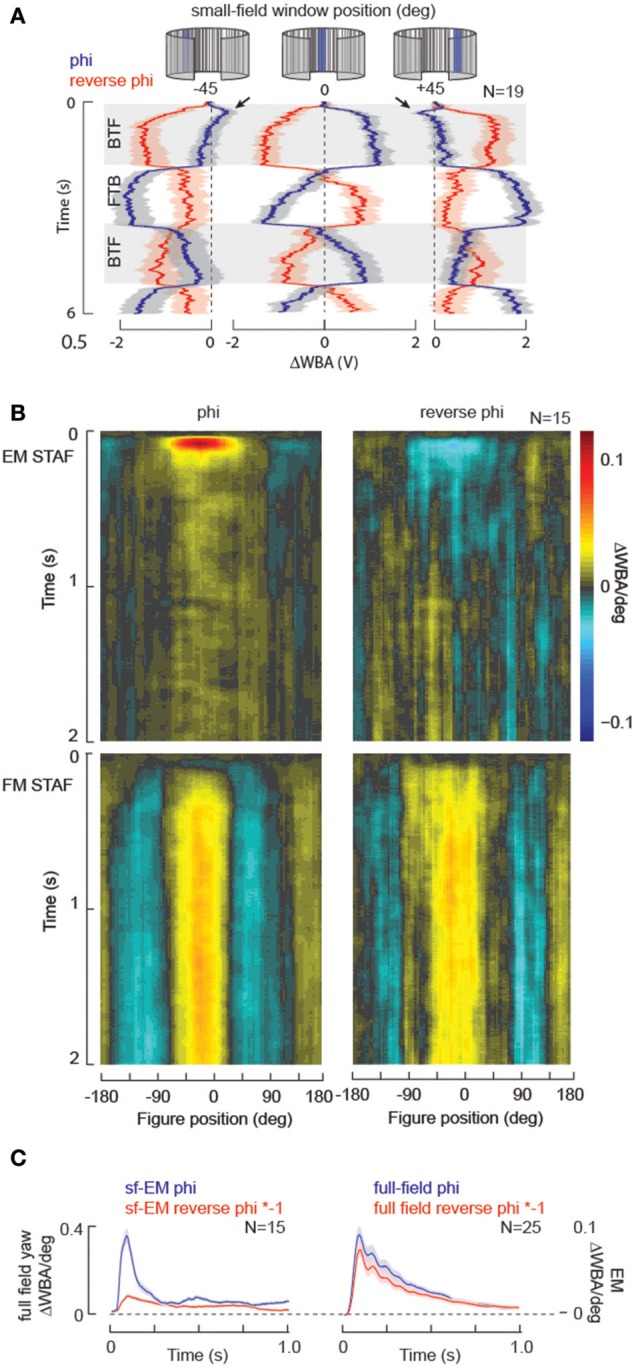
**STAF validation: reverse-phi illusory motion**. For a periodic stimulus, reversing the contrast polarity of the pattern during apparent motion generates the illusion of motion moving in the opposite direction for any motion detection system based on the EMD. **(A)** Data replotted from Figure 4 for normal phi motion (blue), superimposed with results from reverse-phi stimuli (red) collected in the same animals. **(B)** STAFs collected with normal phi apparent motion compared to those collected with reverse-phi stimulation in the same group of individual flies. Note that the EM-STAF is negative, indicating the reverse-phi illusion, but the FM STAF is essentially unaffected by the motion illusion. **(C)** Full-field yaw kernels measured for phi and reverse-phi motion collected from the same flies. By comparison, the “slices” of the EM STAFs at zero-degrees azimuth for the normal phi and reverse-phi stimuli are not equal in amplitude (arrowhead).

Visual systems that compute motion from space-time luminance correlations sampled at neighboring receptors are susceptible to a visual illusion called reverse-phi (Anstis, 1970). For example, a black and white vertical grating pattern that is displayed on a computer screen, drifting to the right is perceived to instead drift to the left if the contrast polarity flickers (black to white and visa-versa). Virtually every animal, including humans, that perceives apparent motion is susceptible to the reverse-phi illusion. The standard implementation of the Hassenstein-Reichardt elementary motion detector (HR-EMD) (Hassenstein and Reichardt, 1956) is also susceptible to this illusion, which provides strong evidence for this model in the computation of motion in biological vision (Aptekar and Frye, 2013), particularly in flies (Tuthill et al., 2011). Proof positive of the primacy of an EMD circuit to a navigational task is mirror-symmetric reversal of an animal's steering effort to a “reverse-phi” stimulus relative to a “phi” stimulus (Figure 4). Furthermore, for the normal phi motion stimuli, the responses to motion in each of two opposing directions are equal in magnitude and time course (Figure 4). Under the same constant-velocity stimulus conditions used to evaluate response symmetry, we tested reverse-phi motion responses in the same flies, which confirms prior results demonstrating opposite directional steering responses (Figure 5A) (Tuthill et al., 2011).

Accordingly, the EM STAF is sign-inverted for the reverse-phi stimulus (Figure 5B). However, consistent with our model of figure-motion (FM) being an EMD-independent quality of a figure-like input, STAFs collected with reverse-phi stimuli reveal that the FM stream is entirely insensitive to the reverse-phi illusion, showing similar spatial and temporal properties for phi and reverse-phi conditions in the same flies (Figure 5B). These results are consistent with a model of figure detection that is described as “flicker dependent” as both a phi and reverse-phi figure on a stationary ground contain similar flicker signals [We note, however, that a flicker-based model fails to explain figure-tracking on a moving ground when both figure and ground contain similar local flicker (Fox et al., 2014), or when the figure and ground flicker at the same rate (Theobald et al., 2010b)].

However, we also note that while, as predicted by the EMD model, the EM STAF shows an inversion of its kernel, consistent with a reversal of the perceived direction of motion encoded by the EM within the figure (Figure 5B), the response is not equal and opposite to the phi response (Figure 5C). This may be expected for some range of pattern velocities because the reverse-phi version of a stimulus tends to flicker at approximately 2x the rate of the complementary phi stimulus (Tuthill et al., 2011). To examine this idea we recorded full-field yaw kernels (Theobald et al., 2010a) at the same frame update rate as the STAFs. Wide-field phi and reverse-phi kernels were collected with an identical group of m-sequences to those used for the STAFs, and the wide field version of the EM response is near perfectly inverted (Figure 5C). Taken together, these results would suggest that the output of EMDs integrated for tracking elementary motion within a moving figure is treated differently than standard EMD-based motion processing implemented within the wide-field motion pathway, and may be worthy of further exploration. This example highlights the power of the STAF technique to identify nuanced differences in the combinatorial processing of multiple motion-cues simultaneously.

#### STAFs to assess eye occlusion and binocular overlap

A useful application of the STAF methodology is to interrogate visual field-specific deficits that may be imposed by limited genetic lesions. Such experiments place stringent requirements on a behavioral assay to be both highly sensitive—able to identify small lesions—and precise—able to repeated across a number of a subjects to similar effect. To validate that the STAF methodology is able to identify such retinotopic deficits, we undertook a set of experiments where we painted over one eye in adult female wildtype *D. Melanogaster* before compiling STAFs for these flies. Animals were tethered to tungsten pins and head-fixed with dental acrylic. Once tethered, while still under cold anesthesia, an eyelash brush was used to apply two coats of water diluted acrylic paint (Carbon Black, Golden Fluid Acrylics, New Berlin, NY) to the cuticle overlying one or the other eye. To verify total coverage of the eye, each preparation was observed and photographed under a 10x magnification dissecting microscope prior to being run. Subjects were rejected if any part of the occluded eye was visible to inspection or if the paint had entrapped the ipsilateral antenna. Subjects were run through the STAF assay according to standard protocol. While we did not expect that eye painting completely blinds the treated eye, we expected the retinal input to be significantly attenuated.

**Figure 6:**
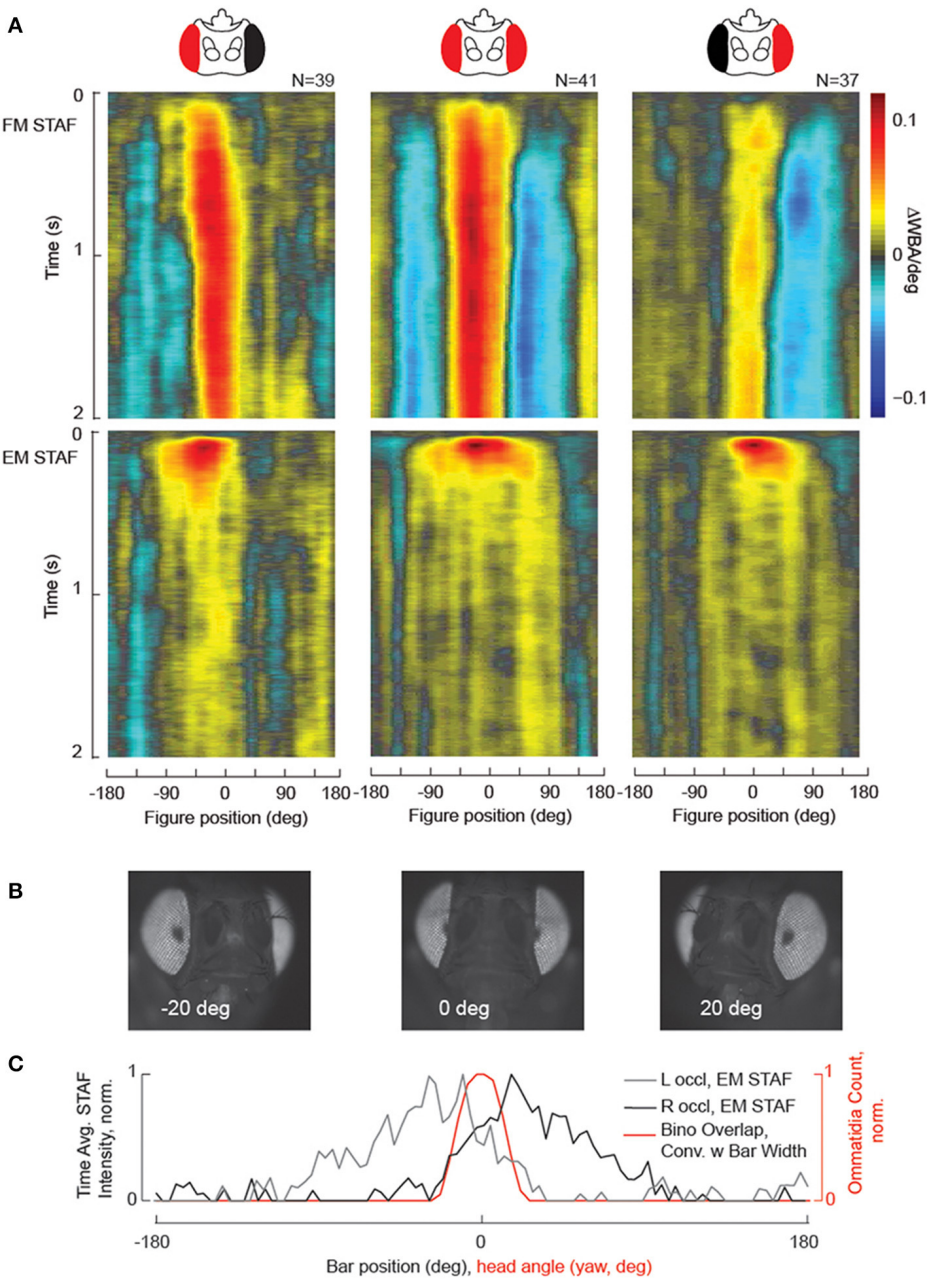
**Localized visual deficits are revealed by the STAFs, and indicate the region of binocular vision. (A)** Black paint was applied to one eye (see Methods for details), which at least partially occluded vision in that eye. FM and sf-EM STAFs are plotted for right occluded flies (left column), intact (center column) and left occluded wild-type flies (right column). **(B)** Images taken from an epi-fluorescent microscope showing the pseudopupil appearing for azimuthal visual angles as indicated. **(C)** The spatial extent of binocular overlap was determined by measuring the size of the pseudopupil appearing on both compound eyes (red) for a single animal imaged across the visual horizon at zero degrees elevation (the ommatidial lattice is similar at each elevation, data not shown). The time-averaged spatial profile of the sf-EM STAFs for the right-occluded flies (gray) and left-occluded flies (black) is superposed with the estimate of binocular overlap. Note that the contralateral extent of the sf-EM STAF coincides with the region of binocular overlap.

Our results clearly demonstrate a significant reduction in behavioral response amplitude in the occluded visual field under the STAF protocol in both the EM and FM channels (Figure 6A). Furthermore, to verify the retinotopic accuracy of the STAF technique, we mounted a fly in two-axis gimbal under our dissecting scope and, using the GFP epifluorescence channel, took photos of the fly pseudopupil over the full azimuth and pitch axes (Figure 6B). The pseudopupil is the region of the compound eye that appears dark when viewed from a particular angle due to colinearity of the viewpoint with acceptance angle of the ommatidia. We used a machine-vision algorithm to count the number of ommatidial facets from each eye visible at each point on the sphere and to reconstruct the region of binocular overlap. The fly was restrained and imaged at 10x magnification with coaxial illumination in a dissecting microscope using a DAPI filter set. This produced strong reflectance from the photopigment and made clear the position of the pseudopupil in one or both eyes. We then produced a threshold mask over the pseudopupil to capture its shape. To calculate how many ommatidia it contained, we tessellated this mask over the original image at eight random locations very near to the pseudopupil where the curvature of the eye was approximately the same. Within each of these tessellated windows, we created a binary mask to identify the septa and a watershed algorithm to count the number of disjoint regions in this mask (the number of discrete ommatidia). Finally, we averaged this count across all eight tessellated windows and used that as the final ommatidial count for the pseudopupil from that vantage. We found that, when convolved with the width of the stimulus bar width (30°), the anatomically measured region of azimuthal binocular overlap was in good agreement with the behaviorally measured region of binocular overlap—defined as the overlap between the two single-eye occluded EM STAFs (Figure 6C), and also in agreement with prior measurements using a different method (Wolf and Heisenberg, 1984). The implication here is that the spatial tuning of the STAFs is in part determined by the region of binocular visual overlap, thus forming a sort of “motion fovea” in the frontal field of view.

#### Statistical analysis to compare STAFs across experimental treatments

**Figure 7:**
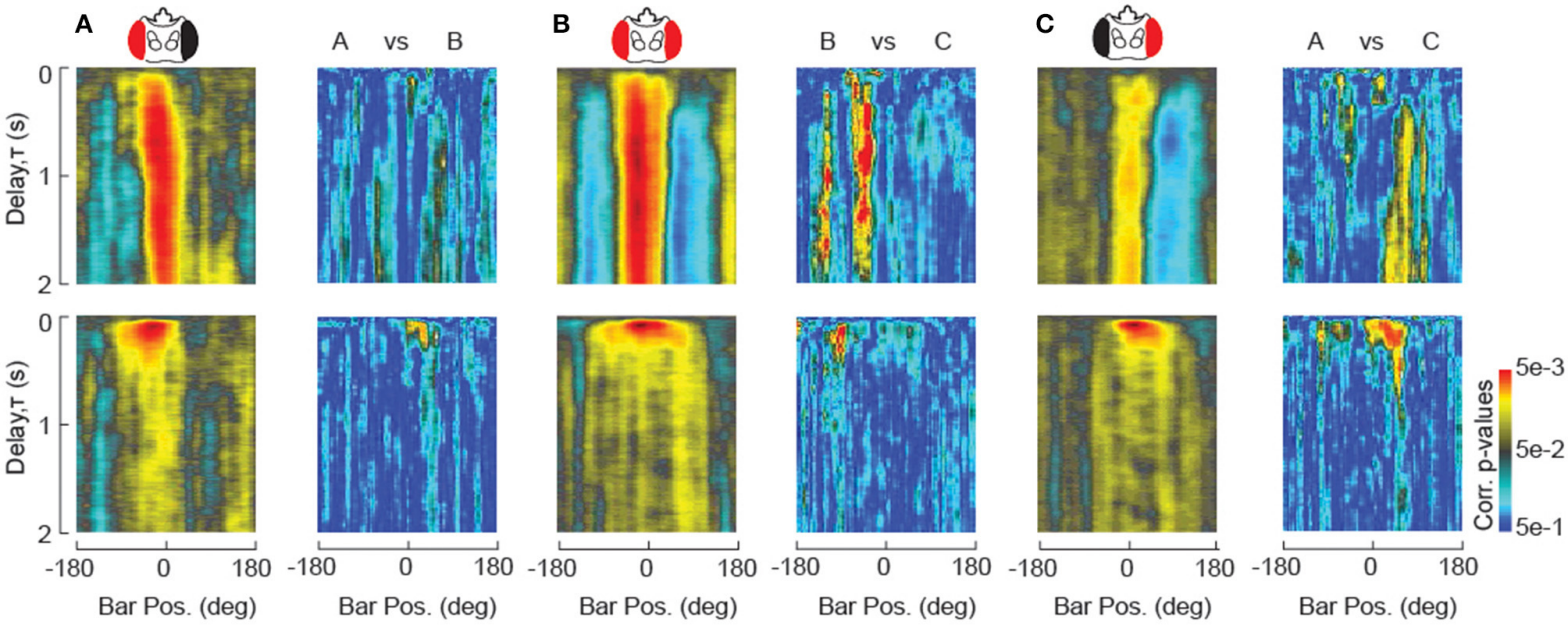
**Statistical comparison of STAFs. (A–C)** STAFs computed for unilaterally occluded flies are re-plotted form Figure 4. For each pair of STAFs as indicated, paired *t*-test measurements at each pixel are plotted in pseudocolor after Benjamini-Hochberg false discovery rate correction (Benjamini and Hochberg, 1995).

In order to establish the general utility of the STAF, it is important to demonstrate that the methodology is sufficiently precise to provide robust statistics for inter-group comparison. This requires enough self-similarity between subjects within a particular group with respect to our method of measurement that groups may be differentiated by a *t*-test or ANOVA. To demonstrate this principle, we provide a set of single-animal STAFs from the eye occlusion study in Figure 6, where one can clearly observe strong features of the average STAF manifest at the level of individual subjects (Figure S1). The dimensionality of the STAF representation is very low with respect to a singular value decomposition, such that a single principal component captures nearly 90% of the population variance (Figure S1), demonstrating that the STAF is in fact a relatively low-dimensional function. This suggests that, although each STAF is composed of ~10^5^ data points, we may significantly correct our false discovery rate (FDR) to reflect this low-dimensionality. We used the Benjamini-Hochberg algorithm to control for the FDR (Benjamini and Hochberg, 1995). This algorithm is suited to control for the FDR in cases where many of the observations (pixels of the STAF) may be positively correlated. Because of the relative large contribution of low spatial and temporal frequencies to the STAFs (i.e., they are relatively smooth), it is suitable to assume a high level of correlation in the values of neighboring pixels and, therefore, the B-H method is well-suited to control for the FDR. Results of the B-H corrected comparisons between the single-eye occluded STAFs are shown in Figure 7. These difference maps demonstrate that the STAF methodology has sufficient precision to provide a robust interpretation of subtle phenotypes resulting from perturbing the underlying circuitry. Animal-to-animal variation is certainly apparent in the STAFs (Figure S1), and analysis of such variation could be facilitated by the STAF method.

Alternatively, to evaluate individual animal performance, a fit-then-compare method to identify significant differences between STAFs is also feasible, as has been deployed to analyze spatio-temporal receptive fields (Woolley et al., 2006). Fitting with a sum of exponentials model identifies the time constants and asymptotic amplitudes of STAFs (Fox et al., 2014) and statistical comparison of the fit coefficients would be more sensitive to small differences that may not reach significance under the pure probabilistic approach given here, and should be employed in cases where the mode of differentiation between test and control groups can be hypothesized *a priori*.

## Discussion

In summary, our work has demonstrated the utility of a white-noise-based system identification technique for analysis of complex, visually-mediated behavior in the fruit fly. In particular, it has painted a clearer picture of two distinct perceptual streams that contribute to figure-tracking behavior:

1. An elementary motion (EM) stream that transduces the space-time correlations in first moment (mean) luminance, presumably via EMD-based processing;
2. A figure motion (FM) stream that transduces higher-order spatio-temporal disparities (e.g., flicker, second moment luminance envelope, and higher-order features such as motion-defined motion) which can be used to signal either the static position or the dynamic movement of a figure, independent of first-order cues; with
3. A total tracking effort approximated by a superposition of the outputs of the two streams.

These results are embodied in Spatio-Temporal Action Fields, a representation that yields a model for optomotor behavior, whose derivation is described in detail in this paper along with the conditions, experimental measures, and limitations required for their validity. We contend that the STAF methodology, when applicable, offers more in this regard than the measurement of raw steady-state responses to the classic repertoire of stimuli—periodic or unidirectional motion of periodic gratings and solid bars—that has been used in past studies of optomotor behavior.

By modifying the STAF methodology, a recent study explored the influence of active figure tracking against a moving visual surround. Instead of displaying separate EM and FM components of a figure on a stationary visual surround, the movement of a solid Fourier bar (EM = FM) and the visual panorama were controlled two m-sequences (Fox et al., 2014). The composite Figure STAF is well approximated by the superposition of the EM and FM STAFS (Aptekar et al., 2012), containing both the rapid EM driven impulse response, and also the slow FM driven step response. The Figure STAF and the Ground STAF show distinct spatial and dynamical characteristics, most importantly demonstrating that the presence of a figure in the frontal visual field either suppresses the normal optomotor response that is driven by azimuthal background motion or that the total control effort is shared by the two subsystems. A potential problem with using the STAF methodology in this manner is that the two m-sequences control EM visual stimuli in adjacent regions of the visual field. The two m-sequences are typically updated at the same frame rate. Thus, for ½ of the total displacements, the figure and the ground are displaced in the same direction by the same amount (a single 3.75° pixel)—the figure, defined here only by its relative motion, would disappear from view. We therefore examined the influence of phase-shifting the displacement of the figure and ground so that the two stimuli are interleaved rather than displaced simultaneously in time. By running these two conditions on the same group of flies, we demonstrated that there is no significant influence of shifting the two m-sequences.

The development and application of the STAF methodology bears significantly on an unresolved dispute in the literature between the view that “position” detection emerges from the D(psi) function (Poggio and Reichardt, 1973), which is based solely on the asymmetry between front-to-back and back-to-front responses to a moving figure, and the view that motion responses are approximately symmetric and position detection is instead based on static receptive fields that are driven by flicker (Pick, 1974, 1976; Buchner et al., 1984). There were two limitations that impeded a broader understanding of the mechanisms at work. First, the temporal dynamics of the two subsystems are crucial to the interpretation, and, prior to our method, there was no way to fully separate the “velocity” component from the “position” component of feature detection *without holding the figure stationary*. A slowly revolving solid bar might generate little flicker but generates other higher-order spatiotemporal statistical disparities that flies track; similarly, a stationary flickering bar is a relatively weak stimulus because it is not moving. By separating the first-order and higher-order properties of a moving visual figure, our prior work generally supports the Pick model, since we deploy low angle displacements (for which no asymmetry can be detected), measure the influence of first-order and higher-order components simultaneously for a *moving* figure, and find that the superposition of the EM and FM components *predict* the Reichardt model responses, *including* the misleading “notch” in the derived motion function (Figure 3). In summary, the EM component is equivalent to a classical “velocity” servo, and the FM component captures a classical “position” servo driven by flicker. However, flicker alone is not the sole determinant of the FM component Theobald et al. (2010b). Instead, other spatio-temporal disparities also contribute.

In more general terms, the decomposition of visual information into visual features is an important function of any high performance visual system. For humans, the field of psychophysics has explored these capacities for more than a century. The evidence from that work points generally to cortical mechanisms for feature extraction. In contrast, a half century of work in flies has shown that these animals accomplish similar feature extraction within the secondary and tertiary optic ganglia—the medulla, lobula, and lobula plate (Egelhaaf, 1985a,b,c; Reichardt et al., 1989; Egelhaaf et al., 1993, 2003; Kimmerle and Egelhaaf, 2000; Aptekar et al., 2012; Fox et al., 2014). As these systems become more tractable with the advent of genetic tools for lesioning and imaging specific subsets of cells within these parts of the fly brain, in addition to the completion of full-fledged wiring diagrams, the need for more nuanced behavioral tools is acute.

The specificity of new genetic tools that robustly and repeatedly target an identifiable cell pathway presents a complementary set of challenges to the behavioral neuroscientist: while it is technically easier to determine the behavioral effects of large lesions to the nervous system of the fly—e.g., the genetic inactivation of many neurons—it is correspondingly harder to identify the functional role of small sets of neurons playing highly specialized roles in visual processing. Lesions that affect few or single neurons may often have only subtle effects on behavior, so that while the identity of the lesioned cells may be well-determined, the behavioral relevance may not be. To overcome these challenges, fine-grained and sensitive approaches to studying behavior are needed.

Because the STAF characterizes both the spatial organization and dynamical properties of an optomotor figure tracking response, it provides a tool for an integrated understanding of the functional components of the visual pathway—and in addition, can help the behavioral neuroscientist who studies genetically targeted lesions to understand where a deficit occurs and what sort of visual processing has been affected. Specific advantages include:

- Retinotopic mapping of behavior: The STAF allows the localization of lesions with respect to a retinotopic location. For visual sensory neurons that sample from sub-regions of the visual field—i.e., have compact receptive fields–lesions confined to a few or single cells will be accordingly limited in spatial effect. Conversely, a spatially extensive effect such as a hemispherical deficit can be identified with neurons that collate information across a broad region of visual space;
- Separation of the effects of several input streams on the visual behavior: The STAF allows attribution of responses to more than one component of a stimulus even within a region of the visual field where the animal responds to these components simultaneously. This technique can be used to identify deficits in the neural circuits responsible for each stream, if they are controlled by distinct neural circuits.
- A measure of system dynamics, or temporal response: The STAF characterizes an animal's dynamical response to each input stream as an ensemble of linear operators or kernel functions. Variations in these kernels can be used to identify subtle effects of targeted ablations of small subsets of cells on the behavior of the animal as a whole;
- And, more generally, as a potential tool for porting animal control strategies into autonomous or semi-autonomous robotic systems, in a format amenable to engineering synthesis and analysis both.

To conclude, this work has demonstrated how two relatively modest innovations on classical white noise analysis—the inclusion of space as a way to organize response kernels and the use of linear decoupling to measure the response to two channels of visual information simultaneously—could substantially improve our basic understanding of the fly visual system. The aim of this paper has been to extend understanding of the STAF methodology by describing the set of behavioral assays and analysis techniques surrounding the STAF formalism in detail, to discuss the particular value of the STAF technique to the study of lesions in the visual system, and to provide relevant software and documentation to facilitate the use of the STAF technique.

### Conflict of interest statement

The authors declare that the research was conducted in the absence of any commercial or financial relationships that could be construed as a potential conflict of interest.
