# Supplementary material for: Method and software for using m-sequences to characterize parallel components of higher-order visual tracking behavior in Drosophila
Source: Front Neural Circuits. 2014 Oct 31;8:130. doi: 10.3389/fncir.2014.00130 (PMC4215624; doi:10.3389/fncir.2014.00130)
Supplement: Supplementary file 1 [file Presentation1.PDF]

Supplementary Material

Review of the m-sequence technique

An m-sequence is a binary-valued series that, although deterministic, has white-noise-like properties; specifically, m-sequences are the shortest, whitest binary sequences with a power spectrum that is identically equal for all frequencies up to the Nyquist limit set by the measurement frequency, with the exception of a dc term that approaches zero for longer sequences (MacWilliams and Sloane, 1976). Characterization of a linear time-invariant system is accomplished by subjecting the system to a train of impulsive excitations made according to a repeatedly-applied m-sequence, and then extracting the impulse response or temporal kernel function from the measured system output by processing that relies on the mathematical properties of the sequence. This technique has been particularly useful in acoustics (Schroeder, 1979; Borish and Angell, 1982; Rife and Vanderkooy, 1989), but has also found applications in a range of other fields in recent years, including, in biology, behavioral reactions to wide-field visual motion in flies (Schnell et al., 2014; Theobald et al., 2010b), reverse-correlation analysis in visual neuroscience (Ringach, 2004), and nonlinear analysis (Benardete and Victor, 1994; Victor, 1992). A clear review of the m-sequence method is given by (Xiang, 1992) and reviewed in Supplementary material. M-sequences were used, as opposed to white noise, because of the binary-nature of them, and because of their relative compactness. As compared to white noise, which asymptotically approaches whiteness for longer sequences, the spectrum of an m-sequence is deterministically white, with the exception of a near-zero DC term.

A binary $\boldsymbol{n}^{\text{th}}$-order m-sequence consists of a string of $\boldsymbol{p = 2^n - 1}$ bits in which every $\boldsymbol{n}$-bit pattern except for $\boldsymbol{n}$ consecutive 0's is represented once without repetition. Such sequences may be generated by a feedback shift-register implementation based on so-called *primitive polynomials* (Golomb, 1968). Conventionally, a binary (0-1) m-sequence is transformed to a sequence consisting of 1's and -1's for purposes of system excitation and identification:

$$\boldsymbol{m_j = 1 - 2m_j^b,\ j = 0 \ldots p - 1}\ , \tag{S1}$$

where $\boldsymbol{m_j^b}$ are the elements of the binary sequence. The arithmetic inverse

$$\boldsymbol{m_j = 2m_j^b - 1,\ j = 0 \ldots p - 1} \tag{S2}$$

of such a sequence is likewise an m-sequence. In addition, any circular shift of an m-sequence (i.e., a sequence that commences with some element $\boldsymbol{m_k}$ other than $\boldsymbol{m_0}$, following the same order and having elements $\boldsymbol{m_0}$ through $\boldsymbol{m_{k-1}}$ appended at the end), as well as order-reversed m-sequences, are also m-sequences. For every $\boldsymbol{n} > 3$ there is more than one *distinct* m-sequence (that is, apart from arithmetic inverses, mirror images, and circular shifts).

The noise-like properties of the m-sequences manifest in its autocorrelation and cross-correlation properties. The circular *autocorrelation* $\boldsymbol{m \star m}$ of a bipolar m-sequence approximates a delta function:

$$\boldsymbol{m \star m}:\ \left(\frac{1}{p}\right)\sum_{j=0}^{p-1} \boldsymbol{m_j m_{(j+k)\ mod\ p}} = \begin{vmatrix} \boldsymbol{1} & \text{for } \boldsymbol{k\ mod\ p = 0} \\ \boldsymbol{-1/p} & \text{for } \boldsymbol{k\ mod\ p \neq 0} \end{vmatrix}$$

or

$$\left(\frac{1}{p}\right)\sum_{j=0}^{p-1} m_j m_{(j+k)\, mod\, p} = \frac{p-1}{p}\delta(k) - \frac{1}{p} \ , \quad \text{(S3)}$$

where $\delta(k)$ is the Kronecker delta. The approximation improves as the order of the sequence increases. Additionally, the circular *cross-correlation* of two distinct sequences, m_j^1 and m_j^2, is small:

$$m^1 \star m^2: \ \left(\frac{1}{p}\right)\sum_{j=0}^{p-1} m_j^1 m_{(j+k)\, mod\, p}^2 \approx \mathbf{0} \ , \quad \text{(S4)}$$

where again the magnitude decreases with sequence order. Finally, *non-stationarity* of m-sequences refers to the non-zero sum of a bipolar sequence, i.e., $\sum_{j=0}^{p-1} m_j = 1$ or $\sum_{j=0}^{p-1} m_j = -1$, depending on whether the sequence is defined by (S1) or (S2), respectively.

The autocorrelation property of m-sequences, along with their whiteness, make them useful for estimation of the impulse response of a linear system, as follows. System excitation is effected by applying a series of positive and negative input impulses according to the sequence, of fixed amplitude and at regular time intervals. The index of the sequence elements corresponds to the discrete times of impulse application. For simplicity, we normalize time by the inter-impulse interval and system input and output by the amplitude of the applied impulses throughout.

Consider the application of two successive periods of an m-sequence, and identifying the time origin with the start of the *second* period, i.e., assume that the stimulus commences at time $t = -p + 1$. Then the system output $y(t)$, which in general is the convolution $y = \int_{-p+1}^{t} g(t-\tau)x(\tau)d\tau$ of the impulse response function $g(t)$ with the input $x(t)$, takes the following values at the times $t = (-p+1) \ldots (p-1)$ at which the impulses are applied:

$$\begin{array}{l}
y(-p+1) = g(0)m_0 \\
y(-p+2) = g(0)m_1 + g(1)m_0 \\
\quad \vdots \\
\boxed{\begin{array}{lllll} y(0) & = & g(0)m_0 & + g(1)m_{p-1} & + \cdots + g(p-1)m_1 \\ y(1) & = & g(0)m_1 & + g(1)m_0 & + \cdots + g(p-1)m_2 \\ \ \vdots & & & & \\ y(p-1) & = & g(0)m_{p-1} & + g(1)m_{p-2} & + \cdots + g(p-1)m_0 \end{array}}
\begin{array}{l} + g(p)m_0 \\ + g(p)m_1 \quad + g(p+1)m_0 \\ \\ + g(p)m_{p-1} + \cdots + g(2p-2)m_0 \end{array}
\end{array}$$

Now suppose that the impulse response dies out within the period of a single application of the m-sequence, i.e., $g(t) \cong 0$ for $t \geq p$, such that the terms involving $g(t)$ at these longer times may be neglected in the equations above − i.e., only the terms in the boxed area need be considered. Then the equations for the responses during the second period can be written in the compact form:

$$y(i) \cong \sum_{j=0}^{p-1} g(j) m_{(i-j)\, mod\, p} \ , \ i = 0 \cdots p-1 \ , \quad \text{(S5)}$$

where the integer index i has been used in place of t to indicate discrete time. The operation represented in (S5) can be regarded a *discrete circular convolution*, and we use the symbol $*$ to represent this operator: $y \cong m * g$.

Now consider the circular cross-correlation u of the m-sequence itself with the system outputs during the second cycle, $u = (1/p)\, m \star y = (1/p)\, m \star (m * g)$. It is well-known that the cross-correlation and convolution operators commute, i.e., $m \star (m * g) = (m \star m) * g$, so that by

applying (S3), the value of $\boldsymbol{u}$ at time $\boldsymbol{i}$ is found to be:

$$\boldsymbol{u}(\boldsymbol{i}) = \frac{\boldsymbol{p}+\mathbf{1}}{\boldsymbol{p}}\boldsymbol{g}(\boldsymbol{i}) - \frac{\mathbf{1}}{\boldsymbol{p}}\sum_{\boldsymbol{j}=\mathbf{0}}^{\boldsymbol{p}-\mathbf{1}} \boldsymbol{g}(\boldsymbol{j}). \qquad \text{(S6)}$$

Thus, if excitation according to an m-sequence is applied for multiple cycles, and the system outputs for times greater than the settling time of the impulse response are considered, then the cross-correlation of the m-sequence with the response values yields an estimate of the impulse response itself. This estimate contains a dc error (proportional to the asymptotic value $\sum_{\boldsymbol{j}=\mathbf{0}}^{\boldsymbol{p}-\mathbf{1}} \boldsymbol{g}(\boldsymbol{j})$ of the *step response*) that decreases with sequence length. In order to obtain an estimate with the same degree of noise rejection, a direct approach to measuring the impulse response – i.e., applying a single impulse, measuring the response out to time $\boldsymbol{p}$, repeating this experiment $\boldsymbol{p}$ times and then averaging the results together – would require $\boldsymbol{p}/\mathbf{2}$ times as long as two applications of the m-sequence. This efficiency is what makes the technique valuable for system identification in flying flies, for which experimental times are limited and noise rejection is important.

Finally, suppose that during an experiment the system output is sampled at a frequency some integer $\boldsymbol{N}$ times greater than the rate of application of image steps. The same cross-correlation methodology can be applied to estimate the impulse response if the m-sequence is padded with $\boldsymbol{N}-\mathbf{1}$ zeros between each non-zero element in the sequence. Note that the upper frequency bound for this method of system identification is therefore set by the Nyquist limit associated with the recording device's sampling rate, given that the m-sequence is transmitted to the fly as a set of near-instantaneous impulses each followed by a finite dwell period.

Fig. S1 The STAF method captures animal-to-animal variation in behavior and is well-conserved across animals. This permits robust statistical comparison between groups. (A) Mean FM STAF for N=16 flies. (B) PC1 for the FM-STAF. (C) Plot of the goodness of fit for the top 40 principle components. Note that PC1 yields >60% higher fit than the next ranking PC. (D) FM-STAFs estimated from single flies.

Matlab code for download. Nine Matlab m-files are available for download, and must be put on the Matlab Path. Please refer to Reiser and Dickinson (2008) for details on construction and programming of electronic visual displays. Each m-file is annotated with comments. "make_6_wide_strip_patterns.m" builds the pattern files to display on the LED display, with each of 48 panels individually addressed (refer to pattern_map within the script for the spatial layout of the address scheme). "make_mseq_functions" produces the m-sequence stimuli to run the experiments. "make_Pattern_FigGrnd.m" builds a related series of display patterns to measure Figure STAF and Ground STAF (Fox et al. 2014). "STAF_Experiment" runs the experimental protocol, and the remaining scripts (fMAKE_STAF.m, fGet_STAF.m, and fPrint_STAF.m) are functions called therein.
