# Supplementary figures and images for: Method and software for using m-sequences to characterize parallel components of higher-order visual tracking behavior in Drosophila
Source: Front Neural Circuits. 2014 Oct 31;8:130. doi: 10.3389/fncir.2014.00130 (PMC4215624; doi:10.3389/fncir.2014.00130)

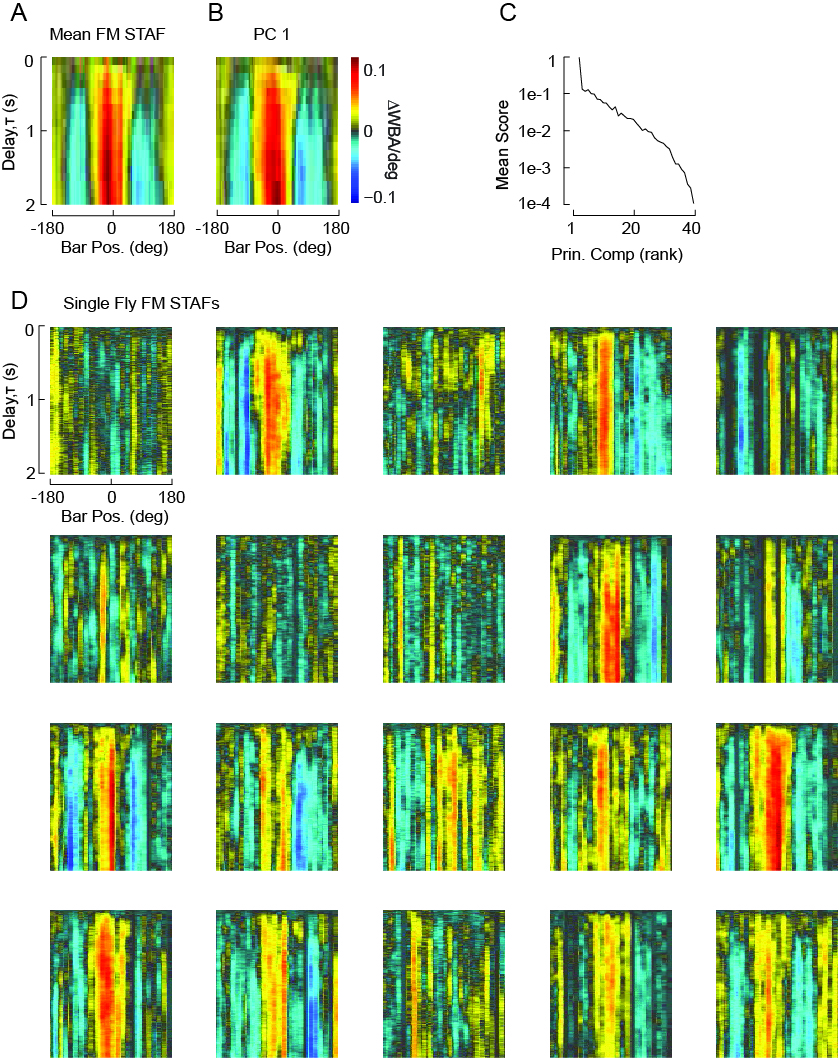

Supplement: Supplementary file 3 [file Image1.JPEG]
